# Pragmatics, Theory of Mind and executive functions in schizophrenia: Disentangling the puzzle using machine learning

**DOI:** 10.1371/journal.pone.0229603

**Published:** 2020-03-03

**Authors:** Alberto Parola, Rogerio Salvini, Ilaria Gabbatore, Livia Colle, Laura Berardinelli, Francesca M. Bosco

**Affiliations:** 1 Department of Psychology, University of Turin, Turin, Italy; 2 Instituto de Informática, Universidade Federal de Goiás, Goiânia, GO, Brasil; 3 Department of Mental Health, A.S.L. "Città di Torino", Turin, Italy; 4 Institute of Neurosciences of Turin, Turin, Italy; National Institutes of Health, UNITED STATES

## Abstract

**Objective:**

Schizophrenia is associated with a severe impairment in the communicative-pragmatic domain. Recent research has tried to disentangle the relationship between communicative impairment and other domains usually impaired in schizophrenia, i.e. Theory of Mind (ToM) and cognitive functions. However, the results are inconclusive and this relationship is still unclear. Machine learning (ML) provides novel opportunities for studying complex relationships among phenomena and representing causality among multiple variables. The present research explored the potential of applying ML, specifically Bayesian network (BNs) analysis, to characterize the relationship between cognitive, ToM and pragmatic abilities in individuals with schizophrenia and healthy controls, and to identify the cognitive and pragmatic abilities that are most informative in discriminating between schizophrenia and controls.

**Methods:**

We provided a comprehensive assessment of different aspects of pragmatic performance, i.e. linguistic, extralinguistic, paralinguistic, contextual and conversational, ToM and cognitive functions, i.e. Executive Functions (EF)—selective attention, planning, inhibition, cognitive flexibility, working memory and speed processing—and general intelligence, in a sample of 32 individuals with schizophrenia and 35 controls.

**Results:**

The results showed that the BNs classifier discriminated well between patients with schizophrenia and healthy controls. The network structure revealed that only pragmatic Linguistic ability directly influenced the classification of patients and controls, while diagnosis determined performance on ToM, Extralinguistic, Paralinguistic, Selective Attention, Planning, Inhibition and Cognitive Flexibility tasks. The model identified pragmatic, ToM and cognitive abilities as three distinct domains independent of one another.

**Conclusion:**

Taken together, our results confirmed the importance of considering pragmatic linguistic impairment as a core dysfunction in schizophrenia, and demonstrated the potential of applying BNs in investigating the relationship between pragmatic ability and cognition.

## Introduction

Communicative impairment has been considered as a core feature of schizophrenia since the condition was first described [1,2], and in recent decades a large body of evidence has confirmed that individuals with schizophrenia (SCZ) show pervasive difficulties in the communicative-pragmatic domain [3–8]. Pragmatic ability can be defined as the use of language and other expressive means, such as gestures, facial expressions and prosody, to act communicatively in a certain context [9,10]. Communicative-pragmatic deficits are frequent in SCZ, resulting in difficulties in the comprehension of non-literal communicative acts, such as indirect speech acts [11] and irony [4,12], and figurative expressions, like metaphors and proverbs [13–15], in understanding deceit [7,16], in recognizing and recovering communicative failures [17], together with deficits in narrative production [18] and conversational skills [19,20]. Pragmatic deficits seem to encompass both comprehension and production, as well as different communicative modalities, such as linguistic, extralinguistic (e.g. gestures), and paralinguistic (e.g. prosody). Patients are, in fact, often impaired in the ability to use and comprehend gestures and prosodic cues to express and recognize communicative intentions and emotional content [8,21,22].

The ability to use language and other expressive means to communicate effectively in social contexts is a high-level process that relies on the complex interaction of different functions, like Executive Functions (EF) and Theory of Mind (ToM) [8,23,24]. However, it is still not completely clear which functions are responsible for communicative difficulties. Recent research has mainly focused on investigating the relationship between communicative-pragmatic impairment and two other domains that are usually impaired in schizophrenia, i.e. ToM and EF. Theory of Mind, i.e., the ability to infer others’ mental states, such as beliefs and intentions [25], has been considered a necessary prerequisite for communication [26–29], and several studies have reported an association between a deficit in ToM and pragmatic difficulties in patients with SCZ in various pragmatic tasks [4,5,14,17,20,30]. Some authors have proposed that a deficit in EF, i.e. the ability to manage goal-directed behavior in a flexible and adaptive way [31], is the primary impairment responsible for the communicative difficulties observed in SCZ, and provided empirical evidence supporting the relationship between EF deficit and pragmatic performance [18,32–34]. However, other studies have indicated no association between pragmatic ability and ToM [14,35], or EF [8,20,36]. A few studies have examined the role of both cognitive functions and ToM concomitantly [8,16,20,37,38]. In a recent study [8] the authors evaluated the role of EF and ToM in explaining the communicative-pragmatic performance of patients with schizophrenia. The results showed the primary role of ToM in explaining patients’ performance in linguistic, but not extralinguistic, comprehension and production tasks, while EFs did not show any significant role in explaining pragmatic performance. Other studies [8,20,37,39] reported an association between ToM and pragmatic ability, even when controlling for the role of EF. However, the nature and strength of this association differed greatly according to the pragmatic tasks used in the different studies. Considered as a whole, the results of previous studies are sometimes conflicting and not conclusive, thus making it difficult to draw firm conclusions about the relationship between pragmatic disorders and cognitive functions in schizophrenia.

Different reasons can explain the variability observed in previous results, and may have prevented to establish robust and reliable findings across studies. Schizophrenia is a highly heterogeneous disorder, with a large natural variability in terms of communicative profiles, clinical features and disease severity. However, previous studies have rarely taken such heterogeneity into account, thus limiting out-of-sample generalizability. A second main issue concerns the very notion of pragmatic ability. Pragmatics involves the appropriate use of a wide range of expressive means, such as language, gestures, prosody, as well as the ability to flexibly adapt our communicative behavior to a specific context, to manage a conversation, to use inferential ability to reconstruct the speaker’s communicative intention. Given the complexity and variety of communicative behaviors, pragmatic ability relies upon different interacting cognitive systems. For this reason, the causal relationship between pragmatic ability and cognition is difficult to define, and the direction of this relationship often unclear. For example, several authors have claimed that ToM is a necessary requisite for developing social communication ability [40–42]; however, experience in and exposure to communicative exchanges may itself improve ToM ability [43]. As a result, cognitive, ToM and communicative factors interact in a complex and bidirectional way, thus often resulting in non-linear and multivariate patterns. Most of the previous studies adopted a classic statistical methodology (e.g., null-hypothesis testing) to explore the relationships between cognitive functions and pragmatic ability. However, this statistical framework has some limitations for representing causality among multiple variables interacting in a complex, non-linear and multivariate way [44,45]. In addition, by focusing more on explaining current measurements than on predicting new data, classical statistical methods may suffer from methodological limitations, such as multicollinearity and overfitting, which may have limited replicability and out-of-sample generalization of previous results.

In contrast, machine learning (ML) methods provide some important advantages over classical statistical methods [44–46]. Indeed, ML uses multiple features to build models aimed at accurately discriminating among different classes—SCZ vs. HC -, and to represent multivariate and non-linear relationships among these variables. Furthermore, the focus on prediction and multivariate analysis offers ML methods greater sensitivity and higher generalization even in case of data heterogeneity. In recent years, several attempts have been made to apply ML techniques to support the diagnostic process in neuropsychiatric and neurological disorders [47,48], identifying the symptom network and predicting the polarity of symptoms [49,50], predicting outcome after stroke and neurological disorders [51,52], representing connectivity between cerebral networks [53], with promising results. These studies showed a higher accuracy in discriminating between patients and control, or in predicting symptomatology, compared to classical univariate techniques (see also [21,54]), thus suggesting a high potential for clinical translation (e.g. to enhance clinical diagnostic and monitoring processes, see also [55,56]).

Among the ML methods, the Bayesian network (BNs) is particularly suitable for representing causality among multiple variables in conditions of high variability, as in the case of investigating the interplay between cognition, ToM and pragmatic ability in clinical conditions like schizophrenia.

### Aims of the study

In the present paper we take a further step beyond previous research [8] to evaluate, for the first time in the current literature, the potential of applying Bayesian network analysis to characterize the functional relationship among cognitive variables, i.e. Theory of Mind and Executive Functions, and pragmatic ability in patients with schizophrenia and controls. We aimed to identify the most informative cognitive and pragmatic abilities in discriminating between patients with schizophrenia and controls, and to represent the interaction among these variables and diagnosis of schizophrenia. We provided a comprehensive assessment of participants’ pragmatic ability, using the Assessment Battery for Communication to identify how the ability to use different expressive means, e.g., linguistic, extralinguistic, paralinguistic, interacts with Theory of Mind and Executive functions.

## Methods

### Participants

The sample of the present study included 32 individuals (25 males) with a diagnosis of schizophrenia according to the Diagnostic and Statistical Manual of Mental Disorders (DSM-IV [57]). The age of the participants with schizophrenia ranged from 22 to 57 years (M = 40.17 years; SD = 10.19); their education level ranged from 8 to 14 years (M = 10.59 years, SD = 2.46). Participants with schizophrenia were all in a chronic phase of the illness and stable in clinical terms: none of them had been hospitalized during the 6 months prior to the study, nor had they undergone any changes to their antipsychotic therapy during the previous 3 months. Their symptomatology was evaluated by an expert clinician with the Positive and Negative Syndrome Scale (PANSS [58]), specifically using the PANSS total score (M = 45.64; SD = 19.02), negative (M = 20.28; SD = 9.65) and positive symptoms (M = 18.83; SD = 8.89) indexes. Cut-off scores were set, as inclusion criteria, on the following neuropsychological tests, in order to rule out any severe cognitive or linguistic deficits: (a) Mini-Mental State Examination (MMSE [59]). Cut-off 24/30; (b) Token Test [60]. Cut-off 5/6; (c) Denomination scale of the Aachener Aphasie test (AAT [61]). Cut-off: no deficit. A group of 35 healthy individuals matched for sex (29 males), age (M = 39.46; SD = 10.95) and education (M = 10.57; SD = 2.46) were recruited. Only Italian native speakers were included. Exclusion criteria for all the participants were: (a) current and/or prior neurological disorder, (b) history of head injury, (c) substance abuse, (d) impaired hearing or vision.

This study was carried out in accordance with the recommendations of ‘A.S.L. To2 ethics committee’ with written informed consent from all subjects. All subjects gave written informed consent in accordance with the Declaration of Helsinki. The protocol was approved by the ‘A.S.L. To2 ethics committee.

### Materials

#### Assessment of communicative-pragmatic abilities

Communicative-pragmatic skills were assessed with the Assessment Battery for Communication (ABaCo) [62–64], a tool designed to evaluate comprehension and production of a wide range of pragmatic phenomena (see Table 1 for a description of the items and Table 2 for mean performance raw scores). The ABaCo is composed of 176 items organized in five evaluation scales, i.e., linguistic, extralinguistic, paralinguistic, context and conversational. For each item, the participants are required to comprehend or produce pragmatically relevant communicative acts either in response to short videos in which two actors play out a communicative exchange, or to vis-à-vis interaction with the examiner.

**Table 1:** Description of the tools administered for the pragmatic and neuropsychological and ToM assessment.

Assessment tool	Brief description
**Communicative pragmatic abilities**	
Assessment Battery for Communication (ABaCo)[62–64]	The *linguistic* and *extralinguistic* scales evaluate the comprehension and production of communicative acts expressed by using linguistic or extralinguistic modality (i.e., gestures or facial expressions). Specifically, the two scales include (a) standard communication acts (i.e., direct and indirect communicative acts), (b) deceits and (c) ironies, expressed verbally on the linguistic scale and through gestures and facial expressions on the extralinguistic scale. In the comprehension tasks, the participants are required to understand the communicative act expressed by two actors in a short clip. In the production tasks, the actors in the clip are engaged in a communicative interaction and, when the clip stops, the participant is required to assume one of the actor’s perspective in replying to his partner. The *paralinguistic* scale assesses the comprehension and production of paralinguistic features, such as prosodic or vocal cues used by a speaker to accompany a communicative act and express emotional contents. Specifically, the scale includes (a) *Basic Speech Acts (statement*, *request*, *question*, *command)*, *in which the participants are required to correctly* comprehend and produce, accordingly, a given type of act based on paralinguistic indicators; (b) *Basic emotions* (anger, happiness, fear and sadness), in which the participants are required to understand (and produce) a particular emotion recognizing (or conveying) a specific emotional tone; (c) *Paralinguistic contradiction*, in which the participants are required to recognize the discrepancy between what is literally said by the actor in the clip and what is expressed via the paralinguistic indicators. The *context* scale evaluates the ability to comply with the norms of social appropriateness and with the conversational rules (i.e. Gricean norms). Specifically, it includes (a) *Grice’s Maxims–* in comprehension only–in which the participants are required to watch a clip and detect and explain the adequacy/inadequacy of the actors engaged in the communicative interaction observed; (b) *Social norms*, in which the participants are require to recognize 8an produce) communicative acts which are appropriate with respect to a given context. The *conversational* scale evaluates the ability to appropriately participate in a conversation with the examiner (lasting 4–5 minutes on a particular topic such as, for example, hobbies), by (a) maintaining the *topic* of the discourse and (b) managing the *turn taking*.
**General intelligence**	
Raven’s Coloured Progressive Matrices[67]	The RCPM is a multiple-choice task based on visual pattern matching and analogy problems, pictured in colored nonrepresentational designs. The participants are required to conceptualize spatial, design, and numerical relations of increasing difficulty. They are presented with a set of incomplete figures to be completed by choosing 1 of the 6 options displayed below the target figure.
**Speed Processing**	
Trail Making test–Part A [68]	The TMT—A consists of 25 circles distributed over a paper sheet. The participant is requested to draw lines to connect the circles (1–25) in ascending order, as quickly as possible. The scoring is based on the time (secs) used to complete the task.
**Selective Attention**	
Attentive Matrices [69]	The participant is shown a series of patterns of numbers displayed on a sheet and they are requested to go through the numbers and find the target on, displayed on the top of the sheet. The task is composed by three sheets of increasing complexity (from 1 to 3 target digits to be found). The scoring is based on accuracy and completion time.
**Executive functions**	
**Working Memory**	
Disyllabic Word Repetition test[69]	The participant is requested to repeat right after the examiner more and more complex sequences of disyllabic words. The sequences range from 1 to 9 words. Scoring is based on the longest series for which 2 or more sequences are correctly repeated.
Corsi’s Block-Tapping test [69]	The participant is shown a set of 9 wooden blocks arranged irregularly. The examiner taps the blocks in randomized sequences of increasing length, (2 to 10 blocks) and the participant is required to repeat the sequence. The scoring is based on the length of the longest sequence where at least two taps were repeated correctly.
**Inhibitory control**	
Modified Card Sorting test[70]	The test consists of four stimulus cards and a number of response cards displaying several symbols, different in color, number, and type of shape. The participant is asked to sort the cards so to place each response card below one of the stimulus cards. Each response card has only one feature in common with three of the stimulus cards, and none with the fourth one. The participants are not given information about the sorting criterion to be used (i.e., shape or color or number), but they are guided to discover the sorting rule. Scoring is based on how many categories were correctly identified and on the number of errors made.
**Cognitive Flexibility**	
Trail Making Test Part B–A[68]	The TMT–B consists of circles containing include either numbers (1–13) or letters (A-L) distributed over a paper sheet. The participant is asked to connect, as quickly as possible, the circles in an ascending pattern, alternating between numbers and letters (i.e., 1-A-2-B…). As well as for Part–A (speed processing), also in part B the scoring is based on the time (secs) used to complete the task. The difference in time used to complete the two parts of TMT (B-A) provides an index of cognitive flexibility.
**Theory of Mind (ToM)**	
**First-order/third person ToM ability**	
Sally and Ann task [71]	The participant is presented 2 paper dolls—Sally and Ann—acting in a false belief scenario. The participant is asked to correctly understand the characters’ behavior on the basis of the characters knowledge and believes.
Smarties task[72]	The task is based on the ‘unexpected content’ paradigm. The participants are shown a box of a famous brand of candies and asked what they believe is in the box. After the participant guesses “smarties”, the examiner shows the actual content is pencils. Then, the experimenter closes the box and asks the participant what another person would think is inside. The task is passed when the participants answer correctly “smarties”, thus showing to be aware of others’ belief.
**Advanced ToM ability**	
Strange Stories tasks [28]	The participant is requested to listen carefully to a number of mentalistic stories (e.g., double bluff, mistakes, white lies…), and answer some questions (e.g., why he/she replied that way?) requiring an inference about the characters’ thoughts, feelings, and intentions. No time limit is given.

**Table 2:** The participants’ mean performance raw scores at pragmatic, neuropsychological and ToM tasks.

Task	Individuals with schizophrenia M (SD)		Healthy controls M (SD)
**Assessment Battery for Communication**			
Linguistic Scale	.79 (.11)	.92 (.05)	
Extralinguistic scale	.67 (.17)	.85 (.09)	
Paralinguistic scale	.62 (.13)	.86 (.08)	
Context scale	.60 (.15)	.91 (.08)	
Conversational scale	.81 (.21)	.96 (.07)	
**General intelligence**			
Raven’s Coloured Progressive Matrices	27.34 (6.42)	33.89 (3.87)	
**Speed Processing**			
Trail Making test–Part A	60.14 (22.22)	33.34 (12.52)	
**Selective Attention**			
Attentive Matrices	44.58 (8.39)	55.55 (4.78)	
**Executive functions**			
**Working Memory**			
Disyllabic Word Repetition test	4.13 (.72)	4.81 (.91)	
Corsi’s Block-Tapping test	4.29 (.82)	5.61 (1.14)	
**Inhibitory control**			
Modified Card Sorting test	.59 (.35)	.89 (.19)	
**Cognitive Flexibility**			
Trail Making Test Part B–A	85.92 (74.35)	32.94 (17.54)	
**Theory of Mind (ToM)**			
**First-order/third person ToM ability**			
Sally and Ann task	.78 (.42)	1.0 (.00)	
Smarties task	.87 (.34)	1.00 (.00)	
**Advanced ToM ability**			
Strange Stories tasks	.65 (.22)	.96 (.09)	

Two independent raters coded the video-recording of the sessions off-line according to the scoring procedure reported in the ABaCo manual. More details about the administration, coding, and structure of the ABaCo are provided in [63] and [65]. The Intraclass Correlation Coefficient (ICC) was used to calculate raters’ agreement, with a result of 0.88, indicating a high value of inter-rater agreement [66].

#### Neuropsychological assessment

To evaluate the participants’ cognitive abilities, we employed a neuropsychological battery assessing the cognitive functions relevant for determining their communicative performance. See Table 1.

General intelligence was assessed with Raven’s Colored Progressive Matrices (RCPM, [67]), speed processing with the Trail Making test (Part A [68]), and selective attention using the Attentive Matrices [69]. We evaluated the following EFs: working memory with the Disyllabic Word Repetition test and Corsi’s Block-Tapping test [69], inhibitory control by administering the Modified Card Sorting test[70]; cognitive flexibility with the Trail Making Test (Part B–Part A [68]). The following tests were used to evaluate ToM performance: First-order/third person ToM with the Sally and Ann [71] and the Smarties tasks [72]. More advanced ToM abilities were evaluated with a selection of the Strange Stories tasks [28]. A description of each task is provided in Table 1, and mean performance raw scores in neuropsychological and ToM tasks are provided in Table 2.

#### Data analysis

To analyze the data we used the Bayesian network approach. Bayesian networks (BN) [73] are probabilistic models that represent a set of variables and their conditional dependencies through a directed acyclic graph. Each node represents an attribute of the domain, and arcs between nodes represent conditional dependencies. For each node it is possible to calculate its conditional probability given the values of their parents. Each variable is assumed to be independent of its non-descendants given its set of parents. Under this assumption the joint probability distribution of all variables can be calculated. A BN can be used for classification when one of the variables is selected as the class attribute, and all other variables are input attributes. For an observation to be classified, the model calculates the posterior probability for each class marginalizing the joint probability distribution and labels the observation with the class that maximizes it.

In this work, the attribute Type of subject was defined as the class attribute, and the input attributes were Sex, Age, Education, Linguistic, Extralinguistic, Paralinguistic, Context, Conversation, General Intelligence, Speed Processing, Selective Attention, Working Memory, Planning, Inhibition, Cognitive Flexibility, and Theory of Mind. The BN model was generated by Weka framework, version 3.8.3 [74]. The Weka learning algorithm used a hill climbing strategy, where several configurations of structures are examined, adding, deleting and reversing arcs between the nodes, such that maximize the likelihood of the class. To generate a simpler and more interpretative model, we set the maximum number of parents of a node as one. The initial count of the simple estimator in order to avoid zero frequencies was set as 0.1. The score type, which determines the measure used to assess the quality of the network structure, was the Minimum Description Length (MDL) [75]. The numeric attributes were discretized by Weka using Fayyad and Irani’s method [76]. We estimated the generalization performance of the BN model using cross-validation, which is a technique for evaluating predictive models by partitioning the original sample into a training set to create the model, and a test set to evaluate it. In our experiments, we used stratified 10-fold cross-validation, where the data is randomly split into ten sub-samples, each one with the same proportion of observations of each class. The process is then repeated ten times and each time a different sub-sample (fold) is used as a test set. Metrics presented are averaged across all folds, and are related to the test sets. We reported the following performance metrics: Accuracy, Sensitivity, Precision and Specificity. Accuracy is the proportion of the total number of classifications that were correct in both classes (SCZ and HC), Sensitivity gives the proportion of cases of schizophrenia classified correctly, Precision gives the proportion of the cases classified as schizophrenia that were correct, and Specificity gives the proportion of cases of controls classified correctly.

## Results

### Bayesian network model

**Fig 1:**
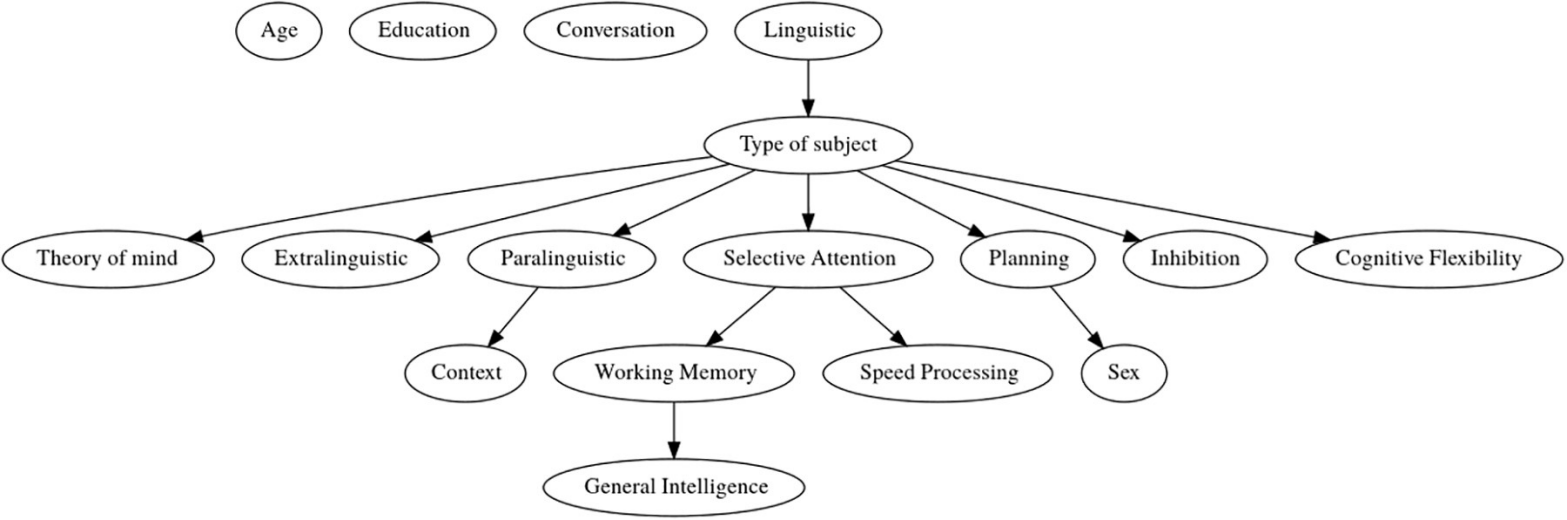
Bayesian network model of pragmatic ability, Theory of Mind and cognitive functions. Each node in the graph represents a variable, and each arc asserts the dependence relationship between the pair of variables. The arc direction tells us that the value of variable X influences the value of variable Y.

Fig 1 presents the BN model structure generated by Weka from the data. It is important to note that not all conditional dependency relationships that may exist between attributes appear in the generated model. This is due to two reasons: the restriction that each node can have at most one parent, and the learning algorithm that sought the best network structure to maximize the likelihood of the class attribute Type of subject. When setting a value to Type of subject (schizophrenia or control), the attributes Linguistic, Theory of Mind, Extralinguistic, Paralinguistic, Selective Attention, Planning, Inhibition, and Cognitive Flexibility become independent of one another. Besides, these attributes are sufficient to calculate the class probabilities for a new individual. Table 3 shows the conditional probability tables of all attributes in the BN model. Thus, we can investigate how the class probabilities are affected by using different attributes, and how much each attribute value is relevant to the classification of a particular instance.

**Table 3:** Conditional probability tables of all attributes in the BN model.

**Type of subject**		
**Linguistic**	**P(Type of subject = “schizophrenia”)**	**P(Type of subject = “controls”)**
**≤** 0.88	0.763	0.237
> 0.88	0.184	0.816
**Theory of mind**		
**Type of subject**	**P(Theory of mind ≤ -3.39)**	**P(Theory of mind > -3.39)**
schizophrenia	0.624	0.376
controls	0.003	0.997
**Extralinguistic**		
**Type of subject**	**P(Extralinguistic ≤ 0.76)**	**P(Extralinguistic > 0.76)**
schizophrenia	0.686	0.314
controls	0.116	0.884
**Paralinguistic**		
**Type of subject**	**P(Paralinguistic ≤ 0.70)**	**P(Paralinguistic > 0.70)**
schizophrenia	0.407	0.593
controls	0.003	0.997
**Planning**		
**Type of subject**	**P(Planning ≤ -1.04)**	**P(Planning > -1.04)**
schizophrenia	0.717	0.283
controls	0.116	0.884
**Selective Attention**		
**Type of subject**	**P(Selective Attention ≤ -0.22)**	**P(Selective Attention > -0.22)**
schizophrenia	0.904	0.096
controls	0.259	0.741
**Inhibition**		
**Type of subject**	**P(Inhibition ≤ -1.03)**	**P(Inhibition > -1.03)**
schizophrenia	0.624	0.376
controls	0.088	0.912
**Cognitive Flexibility**		
**Type of subject**	**P(Cognitive Flexibility ≤ -1.94)**	**P(Cognitive Flexibility > -1.94)**
schizophrenia	0.531	0.469
controls	0.003	0.997
**Context**		
**Paralinguistic**	**P(Context ≤ 0.75)**	**P(Context > 0.75)**
**≤** 0.70	0.992	0.008
> 0.70	0.057	0.943
**Speed Processing**		
**Selective Attention**	**P(Speed Processing ≤ -0.19)**	**P(Speed Processing > -0.19)**
**≤** -0.22	0.055	0.945
> -0.22	0.586	0.414
**Working Memory**		
**Selective Attention**	**P(Working Memory ≤ -0.44)**	**P(Working Memory > -0.44)**
**≤** -0.22	0.840	0.160
> -0.22	0.312	0.688
**General Intelligence**		
**Working Memory**	**P(General Intelligence ≤ -0.62)**	**P(General Intelligence > -0.62)**
**≤** -0.44	0.731	0.269
> -0.44	0.462	0.538
**Sex**		
**Planning**	**P(Sex = female)**	**P(Sex = male)**
**≤** -1.04	0.629	0.341
> -1.04	0.923	0.077

P(X) is the conditional probability of belonging to a class (e.g. Type of subject, schizophrenia or control) or of obtaining a specific value of an attribute (e.g. a value on Theory of Mind below 3.39) given the belonging to a specific class (e.g. schizophrenia or control), or given a specific value on an attribute (e.g. Planning < -1.04) on which it is dependent (according to the dependency relationship shown in Fig 1 represented by a directed arc). As examples: the probability of belonging to the class Schizophrenia is 76.3% given a value below ≤ 0.88 on Linguistic scale of ABaCo, and the probability of having a performance on Theory of Mind ≤ -3.39 is 0.3% given a participant belonging to the class control.

The generated model shows that Linguistic influences Type of subject, i.e., given that Linguistic ≤ 0.88, the probability that Type of subject is schizophrenia is 76.3%. The other attributes are conditional dependent on Type of subject, i.e., given that Type of subject is schizophrenia, 62.4% of the patients have Theory of Mind ≤ -3.39, 68.6% have Extralinguistic ≤ 0.76, 59.3% have Paralinguistic > 0.70, 71.7% have Planning ≤ −1.04, 90.4% have Selective Attention ≤ −0.22, 62.4% have Inhibition ≤ −1.03, and 53.1% have Cognitive Flexibility ≤ −1.94. The most influential attribute in classifying patients with SCZ is Selective Attention (about 90% of the patients have Selective Attention ≤ −0.22). The attributes Planning, Extralinguistic, Theory of Mind, and Inhibition also revealed to be important in classifying patients with SCZ. Patients with SCZ show a large variability in Cognitive Flexibility values (53.1% of the patients have values below, and 46.9% have values above −1.94), probably due to the larger range of values of this test and its complexity (Trail Making Test part B), and thus the attribute is less influential in classifying patients with SCZ. In Paralinguistic patients show a small tendency (of 59.3%) to have values above 0.70, but the attribute is less discriminant compared to Linguistic, Extralinguistic and Context. Other relationships between cognitive and pragmatic variables can be observed in the generated model (see Fig 1). Paralinguistic strongly influences Context. If Paralinguistic ≤ 0.70, the probability of Context ≤ 0.75 is 99.2%, and when Paralinguistic > 0.70, the probability of Context > 0.75 is 94.3%. Selective Attention influences Speed Processing and Working Memory. If Selective Attention ≤ −0.22, there is a high tendency (94.5%) that Speed Processing > −0.19, and a tendency of 84.0% that Working Memory ≤ −0.44. Furthermore, Working Memory influences General Intelligence. If Working Memory ≤ −0.44 is likely that General Intelligence ≤ −0.62, although its values tend to be more scattered when Working Memory > −0.44. Lastly, the probability of Sex is influenced by Planning. If Planning > 1.04, it is very likely that Sex = female.

### Bayesian network model generalization performance

In order to assess the ability of the BN model to generalize beyond the derivation cohort, stratified 10-fold cross-validation was performed. The overall accuracy of the model was 95.5% (SD 7.3%), showing that the model fits well in both classes since they are well balanced (32 cases of SCZ and 35 cases of HC).

As the model classified all cases of schizophrenia correctly, sensitivity was 100%. From all cases classified as schizophrenia, there were three false positives (cases of control classified as schizophrenia), and therefore the precision of the model was 91.4% (SD 12.1%) and the specificity of the model was 91.4% (SD 13.6%) with 32/35 controls classified correctly.

#### Role of clinical feature and medications on cognitive and pragmatic performance

The Bayesian network model described above did not include relevant clinical features (e.g. symptomatology) and medications taken by patients with SCZ since these variables were available only for patient and not for healthy controls.

For this reason, we performed ad hoc analysis in order to investigate the role of psychiatric symptoms and medications on pragmatic and cognitive performance. We split our sample in two subgroups on the basis of severity of symptomatology (low global symptomatology: total PANSS score ≤ 81, and high global symptomatology: total PANSS score > 81). We then built the same Bayesian network model used in main analysis (see Bayesian network model section) trying to predict the belonging of the patients to one of the subgroups (low vs high symptomatology) by using cognitive and pragmatics performance.

The generated Bayesian model failed to classify the severity of symptomatology using cognitive and pragmatic variables. Furthermore, statistical analysis confirmed that there is no correlation (considering the significant level alpha = .05) between these variables according to the Spearman Correlation Test (-0.34 < *r* < 0.1, .052 < *p* < .85).

We also evaluated the role of medications by testing whether cognitive and pragmatic performance was different in patients taking typical and atypical antipsychotic medications. A series of independent samples T-test (considering the significant level alpha = .05) showed no significant differences between the two subgroups of patients (i.e., those taking typical and atypical antipsychotic medications) in cognitive or pragmatic performance (0.43 < *t* < 1.52, .14 < *p* < .97).

## Discussion

A few previous studies have provided a comprehensive assessment of communicative-pragmatic ability in SCZ in order to evaluate the relationship between cognitive functions, ToM [8,20,37,39], and pragmatic behavior expressed through different expressive modalities [7,16,77]. The results of these studies are still inconclusive, potentially due to the large natural heterogeneity in SCZ, the complexity of the multivariate relationship among pragmatic ability, cognition and ToM, and the limitations of classical statistical methods in accounting for these conditions.

ML methods offer the opportunity to overcome such limits and set the stage for an improvement in modeling the relationship between cognition, pragmatic ability and ToM. The present research is the first attempt to apply a Bayesian network analysis in order to identify the complex relationship among the above-mentioned variables in patients with SCZ.

First of all, to evaluate the reliability of the model in classifying patients vs. controls, we measured sensitivity, accuracy and precision. The sensitivity of the model showed that all patients with schizophrenia were classified correctly. The overall accuracy was very high, showing that the model was able to correctly distinguish between patients with SCZ and HC, and precision was also good, showing a very small number of false positives, i.e. HC classified as SCZ.

Given the generated model, the set of variables used to classify a new case consists of the parents, the children and the parents of the children of the class attribute (called Markov Blanket) [74]. Therefore, the following variables are sufficient to discriminate between patients and controls: for pragmatic ability, Linguistic, Extralinguistic and Paralinguistic, for cognitive abilities, Planning, Inhibition and Cognitive Flexibility and, lastly, ToM. However, the structure of the network revealed that only pragmatic Linguistic ability has a direct influence in classifying patients and controls. Indeed, the model showed that a score below the normative value [63] on the Linguistic scale of the ABaCo, indicates a high probability of classification as a patient. We noticed that, of the scales we used during the pragmatic assessment, the Linguistic one best represents pragmatic-communicative ability from an “ecological” perspective, and this might explain why it, and not the other scales, has a direct influence on the classification of patients and controls. This result highlights the importance of assessing linguistic pragmatic ability in SCZ, and is in line with [38], suggesting that an impairment in pragmatic ability could be considered a core dysfunction of the illness. In line with this theoretical perspective, a recent study proposed that communicative-pragmatic deficit could be considered a vulnerability marker of the schizophrenic pathology, and that its assessment, during the early stage of the illness, could improve the diagnostic process [78].

Furthermore, the results showed that the type of participants, i.e. SCZ vs. HC, influences performance on the following tasks: ToM, Extralinguistic, Paralinguistic, Selective Attention, Planning, Inhibition and Cognitive Flexibility. More in detail, belonging to the schizophrenia group was found to be associated with poor performance on ToM, Selective Attention, Extralinguistic, Planning, and Inhibition tasks. A less clear association was found for Paralinguistic and Cognitive Flexibility, where patients obtained a wider range of performance values. These results are in line with a consolidated area of research, starting from Frith’s pioneering proposal [3], showing that patients with schizophrenia suffer from an impairment in mindreading ability [79]. Difficulties in planning, inhibition and cognitive flexibility are also well documented in SCZ [80–82], and our results confirm the importance of including all these functions in the assessment and rehabilitation programs aimed at overcoming patients’ difficulties [83].

Focusing on the relationship among the variables, the model showed that, given the type of subject–SCZ vs. HC—ToM, pragmatic, and cognitive abilities represent three distinct domains independent of one another. This is in line with previous studies [8,38] that, analyzing the pattern of co-occurrence of ToM, cognitive and pragmatic impairments in schizophrenia, pointed out that there is no stable relationship between these functions. This finding supports the idea that pragmatic and ToM deficits cannot simply be reduced to cognitive or ToM deficits [27].

Finally, Education, Age and Conversation are not linked to any other variable in the generated model, which means they are totally independent. The psychometric properties of the Conversational scale of the ABaCo are lower than for the other scales [62], therefore we can assume that this scale did not have a role in discriminating between SCZ vs. HC. As for Education and Age, this result is not surprising, since we kept these variables under control in matching the control and patient groups.

However, it is important to highlight that not all the relationships that could exist among the investigated variables necessarily appear in the generated model. The learning algorithm used a strategy to select from among the possible models (BN structures) based on specialized scores for classification, i.e., to distinguish between patients and controls. Besides, we limited each variable to have at most one parent, that is, only one direct relationship between two variables was allowed (e.g. A → B, but not A → B ← C), to enable the model to choose the most relevant relationship between two variables from among all the possible ones. By using these parameter settings, we were able to find the simplest effective network structure in discriminating between SZ and HC, and we improved model interpretability.

When assessing the relationships among cognitive and pragmatic variables, the model pointed out that performance on the Paralinguistic scale influences performance on the Context one. This result is not surprising, since the Paralinguistic scale assesses the ability to handle cues, such as tone of the voice and prosody that usually accompany a conversation, and that could indicate the real communicative intention of the speaker. The Context scale assesses the ability to comprehend the appropriateness of a speech act in a given situation, and in the pragmatic tasks administered, i.e. short video-clips, the paralinguistic elements are the most important elements enabling participants to detect the social inappropriateness of a proffered utterance (e.g., in an office the boss asks to the secretary to type a letter, and she answers—in a very impolite tone of voice- that she is very busy at moment).

Furthermore, the model showed that Selective Attention influences Working Memory and Speed Processing, and Working Memory influences General Intelligence. In line with recent literature, this result confirms the direct influence that Selective Attention exerts on Working Memory, and the strong associations between these functions [84]. Speed processing and selective attention were evaluated with similar tasks in our sample, both of which require participants to identify the correct stimuli from among several distractors, and the association between these tasks might thus be explained by the task structure. Finally, working memory capacity and intelligence have also been demonstrated to be strongly correlated, and our result provided additional evidence supporting this association [85].

The study is not exempt from limitations: the most important of these is the relatively small sample size, considering that BNs benefit from large sample sizes in order to perform an in-depth investigation of complex dependencies in uncertain data. Future research, with larger samples, will allow the generalizability of the present findings to be assessed.

To conclude, despite its limits, the principal novelty of the present investigation consists in the use of a ML approach to show that, among the cognitive, pragmatic and ToM variables investigated, pragmatic Linguistic ability appears to be the most important factor in classifying patients with SCZ and HC. Communicative-pragmatic difficulty has been associated with a reduction in quality of life, early age of onset, and poor response to treatment [38,86,87], and can thus contribute to the poor social functioning exhibited by patients with schizophrenia. The identification of communicative difficulties as an important factor in predicting SCZ may contribute to improving and refining existing rehabilitative training [88,89,90], psychoeducational programs and clinical interventions aimed at reducing relational and social impairments typical of the illness. However, our results also confirm the role played by other factors, i.e. EF and ToM, in explaining the SCZ profile, and highlight the importance of considering all of these elements at the same time and, at least, with the same level of attention, in order to manage the condition more effectively.

As a final point, we have demonstrated the potential for using BNs to identify the causal influence of pragmatic and cognitive variables in SCZ. This approach enabled us to intuitively represent the network of connections between cognition and pragmatic ability in SCZ, which helped us to identify the most important and clinically relevant variables. The advantages of using ML methods, may lead to important opportunities for advancements in this research field.
